# Facile Fabrication of Porous Adsorbent with Multiple Amine Groups for Efficient and Selective Removal of Amaranth and Tartrazine Dyes from Water

**DOI:** 10.3390/ma17102391

**Published:** 2024-05-16

**Authors:** Qingli Chen, Jie Liao, Sihua Zeng, Li Zhou

**Affiliations:** 1 Guangxi Colleges and Universities Key Laboratory of Natural and Biomedical Polymer Materials, College of Materials Science and Engineering, Guilin University of Technology, Guilin 541004, China; 2 Guangxi Key Laboratory of Calcium Carbonate Resources Comprehensive Utilization, College of Materials and Chemical Engineering, Hezhou University, Hezhou 542899, China

**Keywords:** dye removal, porous adsorbent, glucose, amaranth, tartrazine, selective adsorption

## Abstract

The development of an advanced dye adsorbent that possesses a range of beneficial characteristics, such as high adsorption capacity, swift adsorption kinetics, selective adsorption capability, and robust reusability, remains a challenge. This study introduces a facile method for fabricating an amine-rich porous adsorbent (ARPA), which is specifically engineered for the adsorptive removal of anionic dyes from aqueous solutions. Through a comprehensive assessment, we have evaluated the adsorption performance of ARPA using two benchmark dyes: amaranth (ART) and tartrazine (TTZ). Our findings indicate that the adsorption process reaches equilibrium in a remarkably short timeframe of just 20 min, and it exhibits an excellent correlation with both the Langmuir isotherm model and the pseudo-second-order kinetic model. Furthermore, ARPA has demonstrated an exceptional maximum adsorption capacity, with values of 675.68 mg g^−1^ for ART and 534.76 mg g^−1^ for TTZ. In addition to its high adsorption capacity, ARPA has also shown remarkable selectivity, as evidenced by its ability to selectively adsorb TTZ from a mixed dye solution, a feature that is highly desirable for practical applications. Beyond its impressive adsorption capabilities, ARPA can be efficiently regenerated and recycled. It maintains a high level of original removal efficiency for both ART (76.8%) and TTZ (78.9%) even after five consecutive cycles of adsorption and desorption. Considering the simplicity of its synthesis and its outstanding adsorption performance, ARPA emerges as a highly promising material for use in dye removal applications. Consequently, this paper presents a straightforward and feasible method for the production of an effective dye adsorbent for environmental remediation.

## 1. Introduction

Industrial effluents containing dyes are a significant contributor to water pollution, given the extensive use of dyes across various sectors, including the printing, textile, leather, plastics, coatings, food, and paper industries [1,2,3,4]. Globally, it is estimated that around 1 million tons of dyes are produced annually, and approximately 5–15% of these dyes end up being discharged into water bodies [5,6]. Dye molecules are notoriously resilient to degradation and are often associated with carcinogenic and mutagenic risks, posing a substantial threat to both the ecosystem and human health. Consequently, it is imperative to treat wastewater containing dyes before it is discharged into the environment [7,8]. Previous reports have highlighted common methods for wastewater treatment, including physical adsorption, chemical adsorption, and biological adsorption [9,10,11]. These methods involve techniques such as precipitation, oxidation, electrolysis, photocatalytic degradation, electrochemical degradation, encapsulation, adsorption, and microbial degradation [12,13,14,15,16,17,18,19,20]. Among these techniques, adsorption technology is the most economically feasible, as it offers low cost, high efficiency, ease of operation, wide adaptability, the lowest energy consumption, and environmentally friendly by-products [21,22,23,24,25]. However, conventional adsorbents like activated carbon have their limitations, including low adsorption capacity, limited selectivity, slow adsorption kinetics, and suboptimal reusability. There is a pressing need for the development of advanced adsorbents that can offer rapid adsorption rates, high adsorption capacities, superior selective adsorption capabilities, and excellent reusability. This demand drives ongoing research and innovation in the field of adsorbent materials to address the critical issue of dye contamination in water bodies effectively.

Anionic dyes are as prevalent in industrial applications as cationic dyes, yet the majority of dye adsorbents reported in the literature are primarily designed to target cationic dyes [26,27,28]. This emphasis can be attributed to the fact that most commonly studied adsorbents possess oxygen-containing functional groups on their surfaces. These groups often acquire a negative charge when in aqueous environments, which allows for the adsorption of positively charged cationic dye molecules through electrostatic interactions. In this regard, a variety of natural biomasses, such as bean gum polysaccharides [29], guar gum [30], chitosan [31], pectin [32], and cellulose [33], have been extensively utilized as adsorbents to capture cationic dye molecules from water. On the other hand, porous materials have shown unique advantages in dye adsorption due to their large specific surface area and channels that facilitate dye molecule transport, such as fast adsorption rates and high adsorption capacities [34,35,36]. To effectively remove anionic dyes from water, a promising approach involves the development of adsorbents that combine both porous structures and a positively charged surface. However, despite the potential, the creation of straightforward and efficient methods for preparing adsorbents capable of effectively adsorbing anionic dyes remains in the early stages of development.

**Figure 1:**
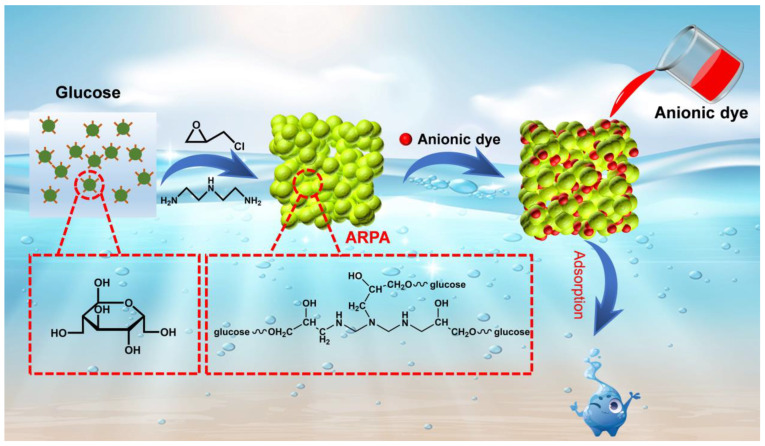
Scheme illustration of the preparation of ARPA from glucose for adsorptive removal of anionic dye from water.

The development of adsorbents that can selectively and efficiently capture anionic dyes is a critical area of research, as it addresses a significant gap in current water treatment technologies. The challenge lies in designing materials that can overcome the electrostatic repulsion between the anionic dyes and the typically negatively charged surfaces of most adsorbents. Innovative strategies are needed to synthesize adsorbents with tailored surface properties that can facilitate the selective adsorption of anionic dyes, thereby contributing to more sustainable and effective water purification processes. The aim of this study is to present a straightforward one-pot synthesis strategy using glucose to fabricate an amine-rich porous adsorbent (ARPA), as depicted in Figure 1. The ARPA is synthesized by reacting glucose with epichlorohydrin (ECH) and diethylenetriamine (DEA). In this process, DEA not only introduces amine groups for crosslinking with glucose but also serves as an organic catalyst to facilitate the crosslinking reaction. The resulting ARPA features positively charged amine groups and a porous architecture, which are highly beneficial for adsorption purposes.

To evaluate the performance of ARPA, two widely used anionic dyes, amaranth (ART) and tartrazine (TTZ), are chosen as model compounds. Both ART and TTZ are commonly utilized in the food industry and have been linked to serious health hazards, including carcinogenic and mutagenic effects. An extensive examination of ARPA’s adsorption capabilities for ART and TTZ has been undertaken, encompassing selective adsorption from dye mixtures, the influence of solution pH and ionic strength on the adsorption process, and the kinetics of adsorption, which measure the rate at which dyes are adsorbed. Further investigation is conducted through adsorption isotherms to elucidate the degree of interaction and affinity between ARPA and the dyes. The thermodynamics of adsorption are also explored to provide a deeper understanding of the spontaneity and energy changes inherent in the adsorption process. To assess the practicality and sustainability of ARPA for continuous use, multiple adsorption–desorption cycles are performed to evaluate its reusability, ensuring its suitability for long-term dye removal applications.

## 2. Materials and Methods

### 2.1. Materials and Chemicals

Glucose (99%), epoxy chloropropane (EC, 90%), diethylenetriamine (DEA, 99%), dimethylformamide (DMF, 99.5%), amaranth (ART), tartrazine (TTZ), methyl orange (MO), Rhodamine B (RB), methylene blue (MB), and methylene violet (MV) are sourced from Beijing Innochem Science & Technology Co., Ltd. (Beijing, China). Deionized water was used throughout the experiments. For reference, Table 1 provides an overview of the molecular structure information of the organic dyes employed in the study.

### 2.2. Characterization

The morphology of the ARPA sample was imaged by field-emission scanning electron microscopy (SEM) (S4800, Hitachi, Tokyo, Japan) and the sample was freeze-dried before imaging. Fourier transform infrared (FTIR) spectroscopy was performed to acquire spectra over the wavenumber range of 400 to 4000 cm^−1^. This was achieved using a Thermo Nexus 470 FTIR (Waltham, MA, USA) spectrometer and KBr disk technique. Elemental analysis was conducted on a Perkin-Elmer 240C Elemental Analyzer (Waltham, MA, USA) to determine the sample’s elemental content. Zeta potential measurements were taken at ambient temperature using a Nanoparticle & Zeta Potential Analyzer from Malvern (Malvern, UK), with pH values spanning from 4 to 10. The absorption spectra of dye solution before and after adsorption were measured on a UV–vis spectrophotometer (Shimadzu, Kyoto, Japan).

### 2.3. Preparation of Amine-Rich Porous Adsorbent (ARPA)

The synthesis of ARPA is simple and can be accomplished through a one-pot reaction process (Figure 1). In a typical procedure, 1.8 g of glucose (10 mmol) was introduced into a solution of 50 mL of DMF and the mixture was vigorously stirred at 90 °C for approximately 30 min to ensure complete dissolution of the glucose. Following this, 3.7 g of EC (40 mmol) was incorporated into the mixture and the reaction was maintained at a stirring temperature of 100 °C for a duration of 1 h to facilitate a thorough reaction. Subsequently, a solution comprising 4.12 g of DEA (40 mmol) and 1 mL of DMF was slowly introduced drop by drop into the existing reaction medium. After addition, the mixture was stirred for an additional 2 h. Finally, the resulting solid was washed with deionized water by filtration, freeze-dried, and the final ARPA product was obtained.

### 2.4. Adsorption Experiments

The adsorption experiment was meticulously designed to evaluate the impact of various factors including pH, NaCl concentration, temperature, the initial concentration of dyes, and contact time. We investigated the impact of pH on the adsorption process by adjusting the pH of the dye solution within the range of 4 to 10 using either 0.05 M HCl or 0.05 M NaOH. The effect of NaCl as an electrolyte on the adsorption efficiency was examined across a concentration gradient of 0 to 1.0 M. To understand the temperature dependence of ARPA’s adsorption behavior, experiments were conducted at temperatures of 275 K, 298 K, and 313 K. Furthermore, the effect of the initial dye concentration, varying from 0.5 mM to 5 mM, on ARPA’s adsorption performance was assessed at a pH of 7 and a temperature of 298 K. The influence of contact time on the adsorption process was also explored under the same conditions of pH 7 and 298 K.

We conducted a typical adsorption experiment, which involved the addition of 20 mg of ARPA and 4 mL of dye solution with a concentration in the range of 1–10 mM into a plastic tube. The mixture was then agitated at regular time intervals until the adsorption equilibrium was attained. To minimize experimental variability, each adsorption experiment was conducted in triplicate, and the mean value was recorded. The concentration of dye in the solution was determined using a UV–vis spectrophotometer. The adsorption performance of ARPA was quantified by calculating the adsorption capacity (Q_e_) and removal efficiency (RE) utilizing the following equations [23,27]:

(1) $$Q_{e}=\frac{{(C}_{0}-{\;C}_{e})V}{m}$$

(2) $$\mathrm{RE}(\%)=\frac{{(C}_{0}-{\;C}_{e})\times 100}{C_{0}}$$

where C_0_ (mg L^−1^) is the initial concentration of the dye, C_e_ (mg L^−1^) is the equilibrium concentration of the dye, V (L) is the volume of the dye solution, and m (g) is the mass of freeze-dried ARPA.

### 2.5. Selective Adsorption Experiments

To evaluate the adsorption selectivity of ARPA for anionic dyes, two binary mixtures were prepared, consisting of TTZ/MV and TTZ/RB. In each mixture, a constant volume of 1 mL of the cationic dyes (MV or RB) was combined with a predetermined concentration of 1 mL of TTZ. These mixtures were then transferred to a plastic bottle and agitated at room temperature for a period of 10 min. Following the adsorption process, the remaining dye concentration in the mixture was quantified using a UV–vis spectrophotometer.

### 2.6. Regeneration Study

Desorption studies were conducted with a 1 M NaOH solution as the eluting medium. Typically, 50 mg of dye-adsorbed ARPA was mixed with 50 mL of the NaOH solution. This mixture was then subjected to a 30 min ultrasonic treatment to facilitate the desorption process. After the ultrasonic treatment, the ARPA was separated from the solution by centrifugation and then collected. The recovered ARPA was thoroughly washed with deionized water until the pH of the wash water was neutral. Once cleaned, the ARPA was freeze-dried for adsorption again.

## 3. Results and Discussion

### 3.1. Preparation and Characterization of ARPA

**Figure 2:**
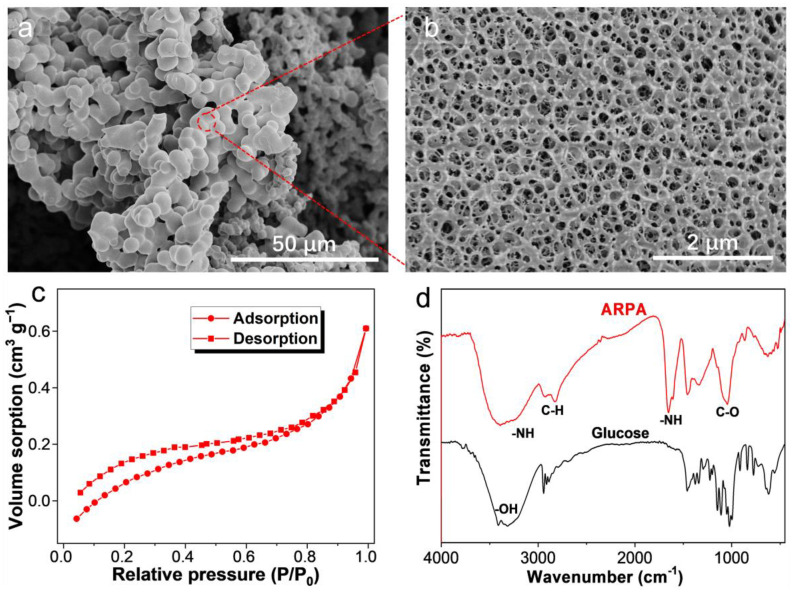
(**a**,**b**) Representative SEM images of ARPA. (**c**) N_2_ adsorption–desorption isotherm of ARPA. (**d**) FTIR spectra of glucose and ARPA.

The synthesis of ARPA is depicted in Figure 1, which begins with the reaction of glucose’s hydroxyl groups with EC, resulting in the formation of epoxy groups. This is followed by the reaction of DEA’s amine groups with the epoxy groups, leading to the creation of the ARPA. In this process, DEA serves a dual role, providing amino groups for the reaction and acting as a catalyst to speed up the process. The morphology of ARPA, as observed through scanning electron microscopy (SEM), is shown in Figure 2a,b. It is evident that ARPA particles, sized between 2 and 8 μm, interconnect through intermolecular crosslinking reactions. Notably, at higher magnification, the SEM image reveals numerous pores within the particles, which are advantageous for the transport of dye molecules during the adsorption process. The specific surface area of ARPA was calculated to be approximately 1.05 m^2^ g^−1^, derived from the N_2_ adsorption–desorption curve presented in Figure 2c. The Fourier transform infrared (FTIR) spectrum (Figure 2d) of the ARPA sample, in comparison to that of glucose, exhibits two significant vibration peaks at 3243 cm^−1^ and 1645 cm^−1^, corresponding to the N−H vibration. This change is attributed to the conversion of glucose’s hydroxyl groups into amine groups through the reaction with EC and DEA. Furthermore, elemental analysis has determined that the N content in ARPA is 15.8 wt%, which indicated the presence of a substantial amount of amine groups within the ARPA structure (about 11.28 mmol g^−1^). Therefore, the ARPA, with its combination of a porous structure and abundant positively charged amine groups, shows significant promise as an adsorbent for removing anionic dyes from aqueous solutions.

### 3.2. Adsorption Performance of ARPA for Different Dyes

**Figure 3:**
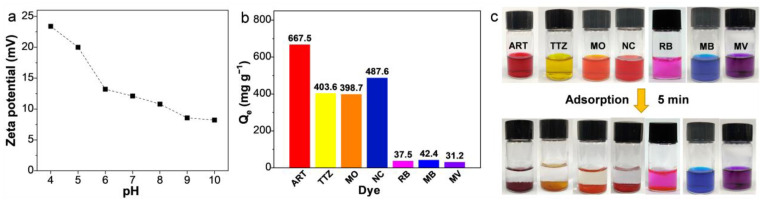
(**a**) Zeta potential values of ARPA at diverse pH values. (**b**) Adsorption capacities of ARPA for different dyes (C_0_ = 10 mM) at pH 7 and 298 K. (**c**) Photographs of various dye solutions (3 mL, 1 mM) before (**top**) and after (**bottom**) mixing ARPA for 5 min.

The surface charge characteristics of an adsorbent play a pivotal role in its adsorption efficacy [37,38,39,40]. To assess this, the zeta potential of ARPA was measured across a pH range of 4 to 10. As depicted in Figure 3a, the zeta potential of ARPA diminishes as the pH rises, yet it remains positive even at pH 10, a phenomenon attributable to the abundance of amine groups. This positive surface of ARPA establishes an advantageous platform for the adsorption of anionic dyes. In order to evaluate the adsorption properties of ARPA, four anionic dyes (ART, TTZ, MO, and NC) and three cationic dyes (RB, MB, and MV) were chosen as test subjects. With an initial dye concentration of 10 mM, ARPA demonstrated significantly higher adsorption capacities for anionic dyes compared to cationic ones after a 15 min adsorption period, as illustrated in Figure 3b. For example, the adsorption capacities for ART and TTZ reached up to 667.5 mg g^−1^ and 403.6 mg g^−1^, respectively, whereas for RB and MB, the capacities were only 37.5 mg g^−1^ and 42.4 mg g^−1^, respectively. At a lower initial dye concentration (1 mM), ARPA was able to remove nearly all anionic dyes within just 5 min, while there was no significant removal of cationic dyes, as shown in Figure 3c. The superior adsorption performance of ARPA for anionic dyes is credited to the presence of positively charged amine groups that engage in electrostatic attraction with negatively charged organic molecules. On the other hand, the adsorption of cationic organic molecules is hindered due to electrostatic repulsion.

**Figure 4:**
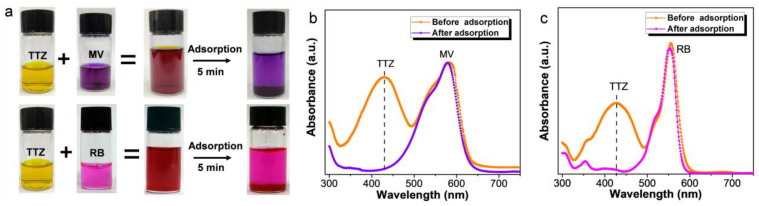
(**a**) Photographs of the dye mixtures of TTZ/MV (0.5 mM) and TTZ/RB (0.5 mM) before and after mixing with ARPA for 5 min at pH 7 and 298 K. The corresponding absorption spectra of TTZ/MV (**b**) and TTZ/RB (**c**) before and after adsorption by ARPA.

In practical adsorption application, a superior adsorbent is able to selectively separate specific molecules within complex aqueous mixtures [41]. To assess the selectivity of ARPA, we conducted adsorption experiments with dye mixtures of TTZ/MV and TTZ/RB. As depicted in Figure 4a, after adsorption, the residual color of the mixture matched that of the cationic dyes (MV and RB), indicating a preferential adsorption of the anionic TTZ dye by ARPA. This selective adsorption process was further monitored using a UV–visible spectrophotometer. Figure 4b,c illustrate that prior to the adsorption, the mixture exhibited two distinct absorption peaks, corresponding to the anionic TTZ and the cationic MV or RB dyes. Upon the addition of ARPA, a significant reduction in the characteristic absorption peak of TTZ was observed, while the peaks for the cationic MV or RB dyes remained largely unchanged. Based on these observations, it can be concluded that ARPA exhibits strong adsorption selectivity for anionic dyes. This selectivity renders ARPA a promising adsorbent for environmental remediation applications, particularly for the removal of anionic dyes from wastewater.

### 3.3. Effects of Initial Dye Concentration, Solution pH, and Ionic Strength

**Figure 5:**
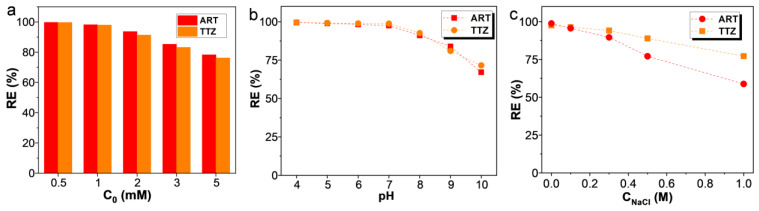
Effects of initial dye concentration (pH 7, 298 K) (**a**) solution pH (C_0_ = 1 mM, 298 K) (**b**) and ionic strength (C_0_ = 1 mM, pH 7, 298 K) (**c**) on the adsorption of ART and TTZ dyes by ARPA.

The adsorption capabilities of ARPA for anionic dyes, specifically ART and TTZ, were thoroughly assessed in a systematic manner. Initially, the influence of the initial dye concentration on the removal efficiency (RE) of ARPA was explored. As indicated in Figure 5a, ARPA demonstrated impressive RE values, surpassing 98% for both ART and TTZ at moderate concentrations (0.5–1 mM). Remarkably, even at a higher initial concentration of 5 mM, the RE remained above 76%, further validating the exceptional removal performance of ARPA for anionic dyes. Recognizing that solution pH is a critical factor influencing the adsorption behavior of adsorbents [42,43,44,45], the impact of pH on adsorption was examined across a pH range of 4 to 10. As depicted in Figure 5b, the results indicate that for pH values exceeding 7, an increment in pH is associated with a corresponding decrease in the adsorption capacity for both ART and TTZ. This trend is attributed to the protonation of amine groups on the adsorbent’s surface at lower pH levels, facilitating the electrostatic adsorption of TTZ and ART dyes. With rising pH, the positive charge density on ARPA’s surface diminishes, as inferred from zeta potential measurements, consequently reducing the electrostatic attraction between ARPA and anionic dyes. Additionally, under alkaline conditions, the presence of OH^-^ ions competes for adsorption sites, further contributing to the reduced adsorption capacity. Consequently, ARPA’s ability to release adsorbed dyes at elevated pH levels becomes feasible. Taking into account the presence of various salts in industrial wastewater, NaCl was selected as a representative inorganic salt to evaluate the effect of ionic strength on ARPA’s adsorption efficacy. As shown in Figure 5c, there is a decrease in ARPA’s adsorption capacity with increasing ionic strength. Nonetheless, even in the presence of 0.5 M NaCl, ARPA can still achieve an RE value of 77% for ART and 89% for TTZ, underscoring its suitability for the practical elimination of anionic dye pollutants in wastewater treatment. Based on the above results, the optimal conditions for the adsorption of ART and TTZ by ARPA are pH 7 and the absence of inorganic salts.

### 3.4. Adsorption Kinetics

**Figure 6:**
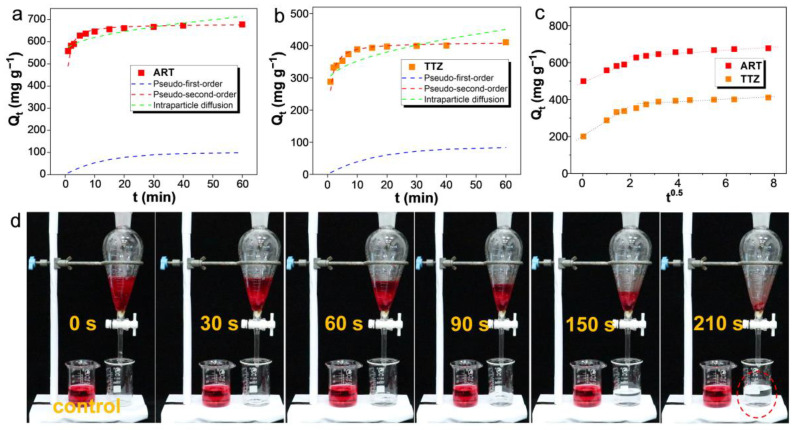
Kinetic fits for the adsorption of ART (**a**) and TTZ (**b**) onto the ARPA (C_0_ = 10 mM, T = 298 K, pH 7). (**c**) The linear dependence of Q_t_ on t^0.5^ based on the intraparticle diffusion equation for the adsorption of ART and TTZ dyes onto ARPA. (**d**) Screenshots of adsorption video based on the addition of 50 mL of ART (1 mM) to pass through an ARPA column. An amount of 150 mg of dried ARPA was utilized to construct the column.

The significance of the adsorption rate is pivotal in real adsorption applications, and thus, the impact of contact time on the adsorption of TTZ and ART dyes was examined. The adsorption experiments were conducted under controlled conditions with an initial dye concentration of 10 mM, a pH level of 7, and a temperature of 298 K. The results, as depicted in Figure 6a,b, reveal that the ARPA possesses an exceptional initial adsorption rate for both ART and TTZ dyes, achieving adsorption equilibrium within about 15 min. The rapid initial adsorption can be linked to the abundance of positively charged adsorption sites on the surface of the ARPA biosorbent. These sites facilitate the initial rapid binding of the anionic dye molecules. However, as time advances, these sites become progressively occupied, leading to a slower increase in the adsorption capacity. To further exemplify the effectiveness of ARPA, taking ART as an example, a column experiment was carried out using 150 mg of the dried ARPA as the packing material, as depicted in Figure 6d. The experiment entailed the passage of a 50 mL ART solution (1 mM) through the column. The collection of a colorless and transparent solution (red dashed circle in Figure 6d) at the end of this process is a clear indication of ARPA’s rapid adsorption rate and high adsorption capacity for anionic dyes. Such characteristics make ARPA an excellent candidate for practical adsorption applications, as it meets the high demands for efficient dye adsorption from aqueous solutions.

To delve deeper into the adsorption mechanism by which ARPA captures anionic dyes, the adsorption data were subjected to a comprehensive analysis using three renowned kinetic models: the pseudo-first-order, the pseudo-second-order, and the intraparticle diffusion models (Figure 6a,b) [46,47,48,49]. The results, as detailed in Table 2, demonstrate a stark contrast in the performance of these models. Specifically, the pseudo-first-order and intraparticle diffusion models produced relatively low correlation coefficients (R^2^), indicating a less accurate fit to the experimental data. In contrast, the pseudo-second-order kinetic model stood out by providing exceedingly high R^2^ values of 0.9991 for ART and 0.9997 for TTZ, respectively. In addition, the calculated adsorption capacity (Q_e-cal_) values (680.3 mg g^−1^ and 411.5 mg g^−1^ for ART and TTZ, respectively) based on the pseudo-second-order kinetic model was close to the experimental adsorption capacity (Q_e-exp_) values (667.5 mg g^−1^ and 404.8 mg g^−1^ for ART and TTZ, respectively). All these results suggest an excellent agreement between the model and the experimental data, indicating that the adsorption process is best described by the pseudo-second-order kinetic model. This model’s applicability implies that the adsorption of anionic dyes onto ARPA is a chemisorption process. The chemisorption mechanism suggests a more stable and stronger interaction between the dye molecules and the ARPA, which is consistent with the high affinity and rapid adsorption rate exhibited by ARPA towards anionic dyes. In addition, the plots of the intraparticle diffusion model, as illustrated in Figure 6c, do not intersect the origin, indicating that intraparticle diffusion is not the sole rate-determining step in the adsorption process. While the intraparticle diffusion model does not provide an optimal fit to the kinetic data, it does delineate two distinct linear phases. The initial, pronounced segment represents the phase of rapid external surface adsorption. This is followed by a more gradual phase, characterized by a subdued slope, which represents the stage where the adsorption process is predominantly governed by intraparticle diffusion.

### 3.5. Adsorption Isotherms

**Figure 7:**
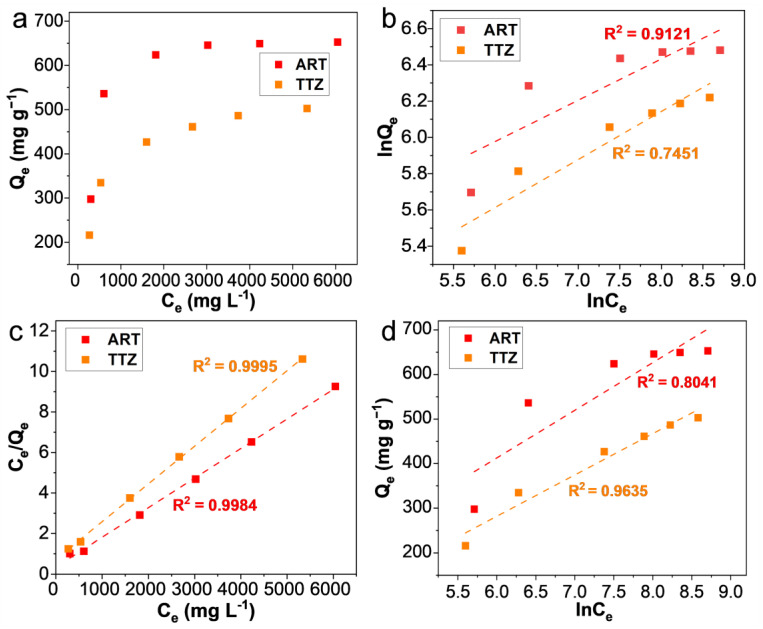
(**a**) Adsorption isotherms for the adsorption of ART and TTZ on ARPA at pH 7 and 298 K. (**b**) The values of lnQ_e_ against lnC_e_ based on the Freundlich isotherm model. (**c**) The values of C_e_/Q_e_ against C_e_ based on the Langmuir isotherm model. (**d**) The values of Q_e_ against lnC_e_ based on the Temkin model.

The adsorption isotherm is instrumental in providing a critical understanding of the adsorption mechanism, as it clearly delineates the equilibrium relationship between the amount of dye molecules adsorbed onto the adsorbent and the residual concentration of dyes within the solution. As depicted in Figure 7a, ARPA displays a significantly enhanced adsorption capacity at higher initial dye concentrations compared to its performance at lower concentrations. This phenomenon can be rationalized by the fact that a more concentrated dye solution promotes the mass transfer of dye molecules from the aqueous phase to the ARPA phase, thereby increasing the efficiency of adsorption. To further analyze the adsorption data, three widely recognized isotherm models were employed: the Langmuir, Freundlich, and Temkin models [50,51,52,53,54,55]. The results, as illustrated in Figure 7b–d and summarized in Table 3, reveal that the Langmuir isotherm model provides the most accurate fit to the experimental data, with R^2^ values of 0.9984 for ART and 0.9995 for TTZ, respectively. These high R^2^ values suggest that the Langmuir model is the most appropriate for describing the adsorption behavior of anionic dyes on ARPA. The applicability of the Langmuir isotherm model implies that the adsorption of anionic dyes by ARPA takes place uniformly at specific binding sites. This process culminates in the formation of a monolayer of adsorbed dyes on the surface of ARPA. This monolayer formation is indicative of a homogeneous adsorption process, where each dye molecule interacts with the adsorbent in a consistent manner.

Based on the Langmuir isotherm model, the determined maximum adsorption capacities (Q_max_) for ARPA with respect to ART and TTZ dyes are found to be 675.68 mg g^−1^ and 534.76 mg g^−1^, respectively. These values are notably superior when compared with a variety of other reported adsorbents (Table 4), such as the zeolitic imidazolate framework (121 mg g^−1^ for ART) [56], chitosan/bentonite composite (362.1 mg g^−1^ for ART) [27], and aminated magnetic polymeric resin (297 mg g^−1^ for TTZ) [57]. This comparison highlights the exceptional adsorptive performance of ARPA.

### 3.6. Adsorption Thermodynamic

**Figure 8:**
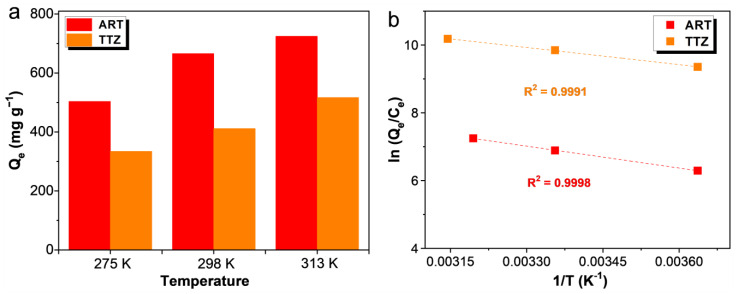
(**a**) Adsorption capacities of ARPA for ART and TTZ at different temperatures (C_0_ = 10 mM, pH 7). (**b**) Plots of lnQ_e_/C_e_ against 1/T for the adsorption of ART and TTZ onto the ARPA.

To ascertain the spontaneity of the adsorption process, the influence of temperature on the adsorption of ART and TTZ dyes by ARPA was studied at three temperatures: 275 K, 298 K, and 313 K. As depicted in Figure 8a, there is a clear trend of the increased adsorption capacity of ARPA with rising temperatures for both dyes, which is indicative of an endothermic adsorption process. Further thermodynamic analysis was conducted using the van’t Hoff equation [58,59], which is expressed as the following:

(3) $$\ln(\frac{Q_{e}}{C_{e}})=\frac{\Delta S}{R}-\frac{\Delta H}{\mathrm{RT}}$$

where Q_e_ (mg g^−1^) denotes the amount of ART/TTZ adsorbed by ARPA, C_e_ (mg L^−1^) signifies the residual concentration of ART/TTZ in the aqueous phase, ΔS (J mol^−1^ K^−1^) represents the entropy change, R (8.314 J mol^−1^ K^−1^) is the ideal gas constant, ΔH (kJ mol^−1^) is the enthalpy change, and T (K) is the temperature in Kelvin. Additionally, the Gibbs free energy change (ΔG, kJ mol^−1^), which is a measure of the spontaneity of the adsorption process, was calculated using the following formula:

ΔG = ΔH − TΔS(4)

The linear plot of ln(Q_e_/C_e_) against 1/T, as shown in Figure 8b, yielded impressive R^2^ values of 0.9998 for ART and 0.9991 for TTZ. Such high R^2^ values indicate a strong correlation and confirm the adherence of the adsorption data to the van’t Hoff equation. The computed thermodynamic parameters, as detailed in Table 5, provide further evidence that the adsorption of dyes by ARPA is an endothermic and entropy-driven process. The calculated values of ΔG suggest that the adsorption is spontaneous, occurring without the need for external energy input, which is a desirable characteristic for practical applications.

### 3.7. Regeneration Study

**Figure 9:**
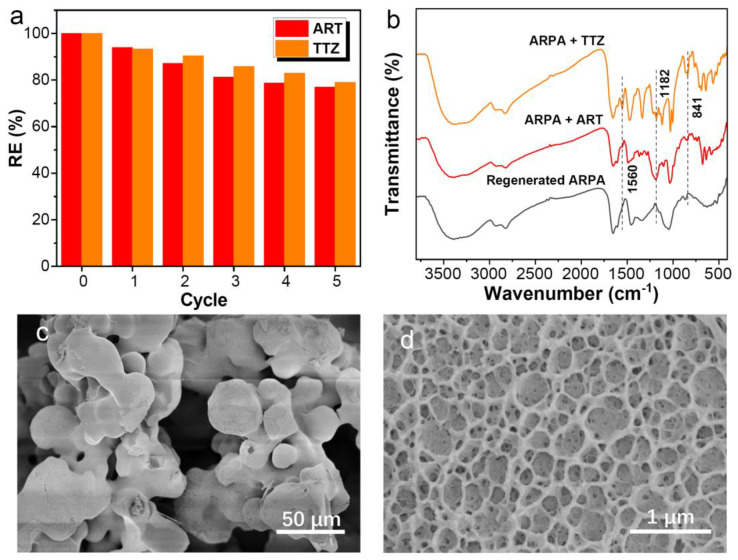
(**a**) Regeneration performance of ARPA in five cycles of desorption–adsorption (C_0_ = 1 mM, 298 K, pH 7). (**b**) FTIR spectra of ARPA after adsorbing TTZ or ART and after regeneration. SEM images (**c**,**d**) of ARPA after being reused five times.

Besides its exceptional adsorption performance, the reusability of an adsorbent is a critical attribute, as it significantly contributes to reducing the overall costs. In this study, the reusability of ARPA was assessed using a 1 M NaOH solution as the eluent to desorb the adsorbed dyes. Following the desorption process, ARPA was recycled and subjected to multiple adsorption cycles. Figure 9a illustrates the reuse efficiency of ARPA, demonstrating that it maintains a relatively high RE for ART (76.8%) and TTZ (78.9%) even after undergoing five consecutive adsorption–desorption cycles. This robust reusability further enhances the practical applicability of ARPA in dye removal. In addition, the FTIR spectra of ARPA after adsorbing ART and TTZ were recorded. As shown in Figure 9b, the distinctive absorption bands of the aromatic ring (e.g., at 1560 cm^−1^ and 841 cm^−1^) and the sulfonic acid group (at 1182 cm^−1^) for both ART and TTZ dyes are clearly discernible. This observation confirms the successful adsorption of the dyes onto ARPA. In contrast, following the regeneration process in a 1 M NaOH solution, the dye-related peaks are no longer present, and the regenerated ARPA exhibits characteristic peaks that are comparable to those of freshly prepared ARPA (Figure 2d). This suggests that the ARPA can be effectively regenerated. Furthermore, the morphology of ARPA after five cycles of reuse was examined using SEM. As depicted in Figure 9c,d, there is a noticeable change in the ARPA particles’ shape when compared to the original, spherical morphology of the freshly prepared ARPA. The particles exhibit an irregular shape after repeated use. Nonetheless, a significant number of pores are still evident on the surface. The persistence of these pores is crucial, as it aids in maintaining the ARPA’s robust adsorption capabilities.

## 4. Conclusions

In summary, our research has successfully showcased a straightforward one-pot synthesis method for creating an amine-rich porous adsorbent (ARPA), which has proven to be highly effective in the removal of anionic dyes from aqueous solutions. The ARPA offers a remarkable combination of attributes: it boasts a rapid removal rate, substantial high adsorption capacity, exceptional selectivity in adsorbing anionic dyes, and commendable reusability. The dye adsorption process by ARPA fitted well with the pseudo-second-order kinetic model and the Langmuir isotherm model. The primary driving force behind the adsorption interaction is identified as electrostatic attraction, which is crucial for the spontaneous and endothermic nature of the anionic dye uptake by ARPA. Taking into account the straightforward production method and the exceptional adsorption capabilities of the ARPA, this work offers a viable approach for the preparation of an adsorbent that is highly applicable for the removal of anionic dyes from wastewater.

## Figures and Tables

**Table 1:** Structure and characteristics of the used dyes.

Dye	Chemical Structure	λ_max_ (nm)	Molecular Weight (g mol^−1^)
ART	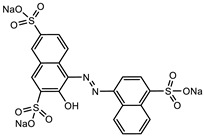	521	604.47
TTZ	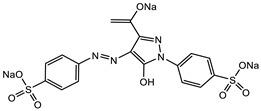	426	534.36
MO	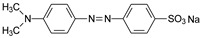	463	327.33
MB	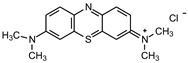	662	319.85
MV	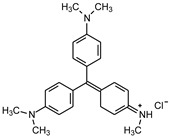	583	407.99
RB	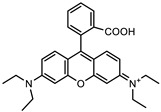	554	479.01

**Table 2:** Adsorption kinetic parameters for the adsorption of ART and TTZ by the ARPA.

Kinetic Model	Parameter	Dye	
		ART	TTZ
Pseudo-first-order: ln(Q_e_ − Q_t_) = lnQ_e_ − k_1_t	k_1_ (min^−1^)	0.0762	0.0613
	Q_e-cal_ (mg g^−1^)	99.96	85.55
	R^2^	0.9538	0.8570
Pseudo-second-order: t/Q_t_ = 1/k_2_Q_e_^2^ + t/Q_e_	k_2_ (min^−1^)	0.0038	0.0042
	Q_e-cal_ (mg g^−1^)	680.3	411.5
	Q_e-exp_ (mg g^−1^)	667.5	404.8
	R^2^	0.9991	0.9997
Intraparticle diffusion: Q_t_ = k_i_t^0.5^ + C	K_i_ (mg g^−1^ min^−1/2^)	20.71	21.55
	C (mg g^−1^)	2.235	4.225
	R^2^	0.7685	0.7124

**Table 3:** Adsorption isotherm parameters for the adsorption of anionic ART and TTZ onto the ARPA.

Isotherm Model	Parameters	ART	TTZ
Langmuir: C_e_/Q_e_ = C_e_/Q_m_ + 1/Q_m_K_L_	Q_m_ (mg g^−1^)	675.68	534.76
	K_L_ (L mg^−1^)	0.0043	0.0027
	R^2^	0.9984	0.9995
Freundlich: lnQ_e_ = lnK_F_ + b_F_lnC_e_	K_F_ (mg g^−1^)	298.2	110.3
	b_F_	0.0908	0.1799
	R^2^	0.9121	0.7451
Temkin: Q_e_ = RTlna_t_/b_t_ + RTlnC_e_/b_t_	a_t_ (L g^−1^)	32.26	0.1811
	b_t_ (J mol^−1^)	45.86	33.28
	R^2^	0.8041	0.9635

**Table 4:** Comparison of the maximum monolayer adsorption of ART and TTZ onto various adsorbents.

Adsorbents	Dye	Q_max_ (mg g^−1^)	Refs
Zeolitic Imidazolate Framework	ART	121	[56]
Fe_3_O_4_@catechol/PEI	ART	146.2	[52]
Graphene/Cellulose/Polyethyleneimine Aerogels	ART	369.37	[53]
Chitosan/bentonite composite	ART	362.1	[27]
ARPA	ART	675.68	This study
GO/CS hydrogel beads	TTZ	293	[54]
SA-GO aerogels	TTZ	420.36	[51]
Aminated magnetic polymeric resin	TTZ	297	[57]
PEI@AC@Fe_3_O_4_-CS/PVA composite	TTZ	148	[55]
ARPA	TTZ	534.76	This study

**Table 5:** Thermodynamic parameters for the adsorption of ART and TTZ onto ARPA.

Dye	ΔH (kJ mol^−1^)	ΔS (J^−1^ mol^−1^ k^−1^)	ΔG (kJ mol^−1^)		
			275 K	298 K	313 K
ART	17.91	117.4	−14.39	−17.09	−18.85
TTZ	8.612	98.20	−18.39	−20.65	−22.62

## Data Availability

Data are contained within the article.
